# Establishment of a multifamily therapy (MFT) service for young adults with a severe eating disorder – experience from 11 MFT groups, and from designing and implementing the model

**DOI:** 10.1186/s40337-020-0285-8

**Published:** 2020-03-02

**Authors:** Tove Skarbø, Steven M. Balmbra

**Affiliations:** Regional Centre for Eating Disorders, Division of Addiction and Specialised Psychiatry, Nordland Hospital, Bodø, Norway

**Keywords:** Eating disorders, Multifamily therapy, Innovation, Implementation, (young) adults

## Abstract

**Background:**

Eating disorders are serious illnesses leading to a substantially lowered quality of life not only for the patient but also for their family. They are difficult to treat, and many patients fail to complete their treatment. At the Regional Centre for Eating Disorders (RESSP) at Nordland Hospital in Bodø, in the north of Norway, it was apparent that many young adult patients maintained an active, ongoing relationship with their family of origin, and that parents and others were often highly involved in their life and illness. It was therefore desirable to develop a treatment model involving family members; specifically a multifamily therapy (MFT) group programme.

**Methods:**

The aim was to establish an MFT service at RESSP for young adult patients suffering from severe eating disorders. This involved, (1) work preparatory to the establishment of the new service, (2) the development and implementation of a suitable MFT model, and (3) sharing the skills and knowledge gained from our experiences to other professionals in the field, and in other settings. This work of development and change can be understood as a process of innovation and is here described within the framework of implementation theory. The work took place in a clinically naturalistic context at the centre.

**Results:**

The MFT model description is based on experience during its development as well as its final form. The stages of this development process and of the changes made in order to establish the new service are described, together with its core components. To date, 68 patients and 198 family members have participated. Dropout rate has been 7.4% and the majority of patients have continued in treatment after completion of the MFT groups. BMI measurements show a significant weight-gain for those with an underweight at start. 32 other professionals have been trained in the model, and a similar service started in 3 other units.

**Conclusion:**

The new model has been welcomed by patients and families alike. The MFT group programme has had a strikingly low dropout rate and a majority of patients have continued in treatment. BMI measurements show a significant weight-gain for those being underweight at start. Other therapists have been trained in the model, and similar services set up elsewhere. In order to document and increase the usefulness of the MFT treatment, a research project has been initiated to evaluate experience and outcomes both quantitatively and qualitatively.

## Plain English summary

Eating disorders are serious illnesses leading to, among other things, a substantially reduced quality of life for both the patient and the affected family. Many patients fail to complete their treatment. At the Regional Centre for Eating Disorders (RESSP) for adults at Nordland Hospital in Bodø, Norway, many parents and others near to the patient were closely involved in their life and illness. On this basis, we wanted to develop a treatment model involving them, through a multifamily therapy (MFT) programme. Work to develop the model and to make the changes necessary to accommodate it, was instigated, and a suitable model was arrived at. The MFT programme has become established as a welcome asset at the centre, and 63 patients and 198 family members and others close to the patient have participated so far. 92.6% of patients have completed their treatment, a strikingly high proportion, and the majority have continued in treatment after the end of the MFT groups. During the course of the groups, the weight of those who were underweight has significantly increased. The MFT model has received recognition by other professionals in the field, and training has been provided to 32 professionals and 3 units have started running comparable services. A research project has been initiated to document and increase the usefulness of the MFT treatment.

## Background

Eating disorders are complex conditions in which genetics, personality, upbringing and culture can all play a role [1–6]. They are severe illnesses which can lead to significant somatic complications and death [7–9]. Eating disorders involve a sharp reduction in quality of life for patients, their families are also considerably affected, and those closest to them are also heavily burdened [10–15]. Poor quality of life is reported among next of kin, and they often require professional help and support in relation to their own emotional responses [13, 16].

Eating disorders are difficult and costly to treat and around 20–30% develop into a severe and long-lasting condition [7, 9, 17, 18]. Studies show that very many patients fail to complete their treatment programme, with a reported dropout rate of up to 50% [18–23]. The relationship between patient and therapist has often been pointed to when attempting to understand and explain this failure to complete treatment [20, 24]. Patients are frequently highly ambivalent with regard to change, and a pattern of insecure attachment can make difficulties in establishing and maintaining an ongoing therapeutic alliance [25–28].

In the treatment of eating disordered children and young people it is customary to include the family. A family perspective is central to, and important in, the treatment and decisive to a good outcome [29, 30]. The latest Norwegian national guidelines relating to eating disorders therefore recommend family-based treatment, tailored specifically to eating disorders, as the first choice at the start of treatment for children and young people [31].

Within the frame of the family-based perspective, a model for multifamily therapy (MFT) for children and young people has been developed for those suffering eating disorders, in particular anorexia nervosa. This is described in the literature, and several treatment manuals are available [32–38]. Regarding adults, the Maudsley Hospital in London has the longest history of including families in the treatment, including MFT groups [39–41]. More recently, Toronto General Hospital in Canada has developed an MFT service for its adult patients [42].

Experience at the Regional Centre for Eating Disorders (RESSP) at Nordland Hospital in Bodø, Norway, showed that many of the young adult patients suffering a severe eating disorder continued to maintain a close relationship with their family of origin, characterized by ambivalence between breaking ties and a ‘need’ for care and support. Parents and other next of kin were often intimately involved in the patient’s life and illness, not least in that the illness can be a matter of life and death. Correspondingly, the eating disorder was often related to problematic family communication and social dysfunction [43], which then represented a hindrance to a favourable developmental process, both for the patient and other family members [27, 44, 45]. Moreover, the families often expressed a need for greater knowledge about the disease, into how they might help and into the stresses they themselves were experiencing [43].

A clear wish to include the families in the treatment offered grew, specifically through the provision of MFT for young adults [43]. The idea by choosing the MFT model was that this model would allow the families to meet in a group situation with space for learning, enquiry and sharing of experiences. The MFT group setting would provide opportunities for gaining new understanding of, and perspectives on, oneself, the members of one’s family and their interactions. There was also the possibility of taking advantage of group analytical factors such as interpersonal learning, socialization, imitative behaviour, universality, and installing hope [46].

The following goals were set: to set up an MFT service for young adults suffering a severe eating disorder and to address the following issues:

- instigate the process of development and change required to establish an MFT service at RESSP
- develop and implement a model for MFT at RESSP
- disseminate skills and experience to other professionals/units throughout the country

## Methods

### The aim of the study

The primary aim of the project was to establish an MFT service for young adults with a severe eating disorder. The challenges were (1) to initiate the development work and to initiate the changes necessary to establish an MFT service, (2) to arrive at, and implement, an MFT treatment model at RESSP, and (3) to communicate our knowledge and experience to other professionals and services nationally.

### Design and setting of the study

This process is best understood as a process of innovation and is described within the framework of implementation theory [47]. Innovation in this context is seen as a process of planned change, with the aim of improving practice [47–49]. From the very first, this work has taken place in a clinically naturalistic context.

### Participants

Participants were patients with a severe eating disorder (anorexia or complex bulimia) and family members interested in taking part in this clinical group process over the course of 12 months. Information was sent out to all the treatment centres throughout the health region. Patients in the age range 18–30 were offered the service. One requirement was that they had not yet established a family of their own and had an ongoing relationship with their family of origin. They also had to belong to the catchment area of the health region and have on-going contact with a therapist in the specialist health services who could provide the necessary follow up.

The family members who were invited to participate were parents (mother and father), siblings (sisters and brothers) and others particularly close to the patient, such as partners, boy−/girlfriend and occasionally close friends.

In addition to the above criteria there was an initial evaluation of the patient and family following their application to take part. A requirement was that all participants were interested in, and capable of taking part in a group with other families and being open to other people’s perspectives and views. Exclusion criteria regarding family members were serious psychopathology, suicidal intent and substance abuse or dependency. These criteria were seen as necessary to allow a healthy group process.

The participating therapists were staff at RESSP. They came from different professions and had differing levels of experience and competence – psychologists, psychiatrists, family therapists, art therapists, psychiatric nurses, social workers and fitness consultants were all represented. They were drawn from both the inpatient unit and the outpatient clinic.

The service at RESSP is for patients 18 years and older, and covers the three northernmost counties in Norway. It consists of an inpatient unit with 12 beds and an outpatient clinic. The centre has 42 staff.

### Description of process, interventions and comparisons

In implementation theory, the processes involved in are grouped together in six phases, which can overlap, and which do not necessarily follow in their given order [47]. These phases are: exploration and adoption, programme installation, initial implementation, full operation, innovation and sustainability.

The main intervention was the choice of change, which involved selecting the changes that were considered the most suitable. The core components of such an intervention – also called implementation drivers – are those seen as being necessary to enable change within an organisation [47, 50, 51]. The components are as follows: recruitment and staff selection, pre-service and in-service training, ongoing coaching and consultation, staff performance evaluation, programme evaluation, facilitative administrative support, systems interventions and decision support and data systems.

Comparison, in the context of this project, refers to an evaluation of the quality of the implementation. Implementation quality gives a picture of the relationship between the planned changes, and those carried out in practice. Quality is measured by the extent to which the changes were carried out in accordance with the original proposal or idea [48]. Models are developed for the evaluation of the quality of implementation. Greenberg et al. [48] are concerned with the discrepancy, or deviation, between the planned intervention and the actual intervention, while Domitrovich et al. [52, 53], in addition to the question of deviation, pose a question as to whether, or not, there is a support system in place for the intervention – things such as supervision and a suitable environment which support the development of the intervention.

### Statistics

Simple, descriptive statistics have been used to give an overview of the number of participants, demography and BMI values at start and finish. Paired-samples t-tests were conducted to compare the BMI at the start and end of MFT, as well as for those with AN and those underweight at start, respectively using IBM SPSS statistics program (version 24). Cohen’s *d* effect sizes for within-sample changes in BMI from start to end of MFT were calculated. (Cohen’s *d* values of 0.2, 0.5 and 0.8 are considered to indicate small, moderate and large effects. A value of 1.2 is considered to indicate a very large effect [54].)

## Results

### Implementation of the MFT service – and the creation of an MFT model for young adults

In this process of innovation there were no right answers to turn to. It was necessary to tailor the proposal in order that it be suited to the patient group, the family members, existing treatment services, the organisation, culture and geography.

We took the model in the MFT training for children and young people in Tromsø as our starting point (for more information see part ‘Elements of the innovation’), together with the multifamily services for young adults with eating disorders in other treatment centres, namely Maudsley Hospital in London and the Toronto General Hospital in Canada [39–42].

It was necessary to make adjustments to the aims, the structure of the gatherings, content, themes and interventions. The main aims were to help the young adults to take responsibility for their own lives and health, and to broaden communication and interaction within the family. A secondary aim was to reduce eating disorder symptoms such as low weight. To attain these goals, participants had to be engaged by, and involved in, the process. The frame of the groups had to be tailored to these considerations.

### On-going experience and the design of the service

When the first MFT group started in 2007 an underlying structure for the service was in place, but all the details were not. On the basis of experience and feedback, and by means of supervision, the service was continually refined and adjusted. After 3 years an apparently well-functioning model, that received good feedback from both patients and families, had been developed. From the fourth group onwards there has been a permanent shape to the service. An MFT handbook was produced [55].

The MFT programme consists of 6 group meetings spanning over 12 months. Each of the gatherings address specific and relevant themes for the families and participants (see part ‘Structure and content of the gatherings – relevant issues’).

### Objectives

The main objective of the MFT group was that the patients would take responsibility for their own lives and health, reducing their dependence on their families of origin. The families that were over-involved would need to be able to step back from, or reduce, their level of responsibility. The main theme was, correspondingly, movement away from an emphasis on the ‘dinner plate’ towards relating, interactivity and communication within the family. Working with specific symptoms of the eating disorder such as low weight was a secondary aim. The idea was that, by means of working with the above mentioned themes, the way in which the family could best support and contribute to the recovery process would be made clearer. It might help to ‘loosen up’ unhelpful patterns that might otherwise serve as factors maintaining the illness. The situation of the siblings was also a concern within the programme, providing room for their voices by addressing one of the MFT meetings to this issue. Another objective was that the families would gain more insight into the eating disorder and explore relevant themes relating to it, including both the stresses and worries of the individuals and families and their resources.

### The framework

The framework was derived from the one developed and tested in connection with the training established in Tromsø. A corresponding programme of 6 gatherings, the first lasting 3 days and the other five 2-days, over the course of 12 months, totalling 13 days, was arrived at, together with an initial half day information meeting. This framework was set to meet practical conditions related to geography and the travelling distances. Because of the intensity of the programme, the participants needed time to recover and integrate.

This framework worked well from the start, and we found no change necessary. The service is now established as a group treatment process over the course of one year.

The number of families able to participate in the MFT groups is between 6 and 8. It is considered that there should be one therapist per family, which means that the number of therapists involved varies in line with the number of families. It was, however, seen as advantageous to have an additional therapist in particularly demanding situations. The number of therapists and co-therapists was seen as useful in supporting the therapeutic work as well as giving the possibility to build a common understanding and competence among the staff.

The therapeutic team consisted of professionals from different backgrounds. Two therapists having overall responsibility for all the MFT gatherings throughout the year, although these could change from year to year. Their responsibility was to plan, and maintain, the planned structure of the gatherings, as well as evaluating the process as it went along. The evaluation was carried out by means of daily meetings among the therapists addressing the therapeutic content of the process, the interaction among the participants and the therapeutic team, the group cohesiveness and the overall therapeutic development.

It was essential to plan and prepare each gathering, and also amidst the groups to allow an overview of the process and for tailoring the content.

### Structure and content of the gatherings – relevant issues

Broadly speaking, the MFT model is built up as a progression in respect of both structure and themes that follow how well the group is established. The themes were chosen based on competence and experience. Usually, individual issues are introduced to the group by means of psychoeducation, after which the issue is worked with in different group settings. At the beginning, there is a great deal of information to deliver to the group, and so a lot of time is set aside for that, with time as well for dialogue and discussion. Later, work starts with the issues more directly, and work with more emotionally demanding topics, such as feelings of guilt and shame are taken up during the later gatherings. Different group settings are used, the large group but also small (peer) groups comprising, variously, mothers, fathers, siblings and patients, as well as groups consisting of just two people.

Certain issues emerged early on, as being especially relevant and of essential value, such as the situation of the siblings and the feelings of guilt and shame, and these were integrated into the programme. We also experienced that the best structure was to give a central theme to each gathering. The following themes were assigned to the different gatherings: (1) Establishing the group – about eating disorders – how to understand them, and what they do to a family – motivation and change and ambivalence. (2) Communication and communication styles (and relationship and interaction within the family – how we relate to each other). (3) Care and patterns of caring – how best to be supportive – belonging. (4) Siblings – focus on their situation - how is it for them? (All participants taking part in this gathering.) (5) ‘Mind the gaps’ – concerning transitions (the ‘home’ therapists of the patients are invited to this gathering). (6) Summing up and ending – the way forward.

During the first gatherings, a firm structure was seen to be necessary, and so each day is divided into parts with clearly defined subjects. It was also found to be worthwhile differentiating between main themes and other, equally relevant, sub-themes. There are issues which reoccur, such as conflict management, guilt and shame, fear and anxiety. Presentations were given about such themes as the medical consequences of undernourishment, motivation for change, patterns of attachment, systemic thinking, mentalising and models for understanding conflicts, and improvement and recovery.

### Therapeutic interventions

Several of the therapists involved in the MFT service had long clinical experience of working with eating disorders and other clinical conditions and had postgraduate training in, among other qualifications, systemic family-therapy, group therapy, psychotherapy, psychodrama and art therapy, all of which was natural to make use of in the MFT work.

Different ways of working were chosen for different topics, based on the background of clinical competence and experience, as well as evidence-based knowledge*.* The work needed to engage both the patient and the family members. (See the MFT handbook for a more detailed description of the various ways of working [55].) The following are used: psychoeducation to inform and educate about the various themes. Family relations and networks are visualised by way of a genogram (family map), spectograms and overviews with the Family Dialogue Set (FDS). Roleplay and role-exchange are used to explore episodes and issues. Panel debates are employed during the ‘sibling gathering’ (the 4th) in which expert panels of patients, siblings and parents are formed. Creative exercises and tasks are used to work on difficult issues in concrete and visual ways. In these exercises, the families draw, model and construct – for example – a collage of life with and without the eating disorder. They make a sculpture of the ‘uninvited guest’ in their family; making models of a ‘tower of guilt’ and ‘tree of aims’. These visual expressions lead to further conversations and exploration. Visualisation techniques are used – different metaphors such as the ‘uninvited guest’ to symbolise the eating disorder, and ‘weight warriors’ or ‘food fighters’ as a symbol of patient with the eating disorder and the difficulties they face. Brainstorming – (especially in the 3rd gathering) – helps to arrive at resolutions to conflicted situations. Pen and paper are used to gather up individual reflections that can be shared in groups later on.

At the end of each gathering, a homework task is given for completion by the next gathering. This is simple, concrete and related to the theme of either the present or the coming group. An example would be for the family to agree to do something practical to improve their situation.

### Group process

To facilitate a fruitful group process, it was necessary to take into consideration both the group as a whole and the characteristics of the different families and individuals. The presence of several families facilitated a group-dynamic process leading to new contributions, challenges and the possibility of ‘playing off’ others through mirroring and resonance. This made space for increased awareness, understanding and change. To create a fruitful group process like this, it is essential to work with the most important and relevant issues. Each individual can meet themselves in this work, and ‘feel it in the body’. To maintain a good dialogue and process in the group it is crucial to know ‘where the group is’. The level of the emotional climate in the group, in the families and in each individual must constantly be ‘read’ and responded to - and held at a desirable level, neither too high (‘warm’) nor too low (‘cold’), but at a level well suited to exploration, understanding, learning and growth. This means balancing the group between challenging and calming. If the group becomes too ‘cold’, the advice from the supervisor was to dare to create small crises which could be worked on. If too ‘warm’, the heat had to be taken out of it. At the end of the work, concluding exercises were employed to round off the group process.

Groups start with plans for managing the particular subjects, but it is necessary, at all times, to be aware of the actual process at play in the group, the families and individuals, and regulate them as required. Group leaders must be flexible in respect of the original plan and bear in mind that individual processes can be triggered by the material and be prepared to manage this.

### About the implementation process – the development work and the changes made in order to establish the MFT service for young adults at RESSP

The implementation started with a unifying shared understanding among the staff, and a shared desire to develop the MFT service. To enable this, they were actively involved in the process from its starting point in 2006 and played a consistent and decisive role in the work.

In the programme installation phase a supervisor was acquired. Sufficient time was set aside for supervision and ongoing meetings. At this stage, the staff showed a loyalty and engagement with the process of change.

In phase three, initial implementation, the staff were both willing and able to persevere through the difficulties and stresses involved in the demanding process.

The full operation phase meant that the new MFT service would be integrated into day-to-day service over delivery and thereby became a part of the organisation. Through time spent on systematic testing and reworking, a model for the MFT work was developed into an established service provision.

Throughout the entire process there was an ongoing evaluation of the new service provision. Minor adjustments were necessary to ensure that the provision was in accordance with the designated aims, and that it suited the participants of each group.

The MFT service has been established for several years and has become a part of the professional profile of the centre. It was possible to reach this position through a sustained focus on the work with MFT and ongoing facilitation of training in, and of inclusion of newly appointed staff in the work.

### Elements of the innovation – core components of the completed development

Regarding staff selection, both clinical and support staff were asked about being involved in the MFT work. On the basis of their background, skills and experience, a number of different professionals expressed a clear interest in participating, and this resulted in a multidisciplinary team with differing approaches and treatment skills.

Many of the therapists had long experience of different kinds of therapies, but none had experience of MFT work which requires clinical competence at both individual and group level, as well as skills in disseminating knowledge. It was therefore necessary to arrange for and set aside time for training and professional development. Three therapists took part in an MFT training (for children and young people) over 1.5 years, delivered by the Regional Centre for Eating Disorders (RSS) in Tromsø by Penny Fairbairn and Ivan Isler from London. Subsequently, two other therapists took part in an MFT training carried out by Ulf Wallin in Lund, Sweden.

Penny Fairbairn was asked to supervise the first stage of the development and implementation work. This shared supervision contributed to the creation of a common understanding of the MFT work. Penny was a valuable support and catalyst in realising the MFT service.

Regarding staff performance, provision was made for ongoing feedback among the therapists, during and after each MFT day and after each gathering, as well as feedback received from the participants. The process was evaluated and thought given to how best to address relevant events within a topic-based programme.

In programme evaluation we had to manage the changes to become part of the unit’s practice and culture. Systematic ongoing verbal evaluations between the therapists were given and received concerning progress towards the stated goals and strategic objectives. Other professionals at the centre shared in the evaluations.

Concerning facilitative administrative support, the leadership gave significant priority to supervision and evaluation and made provision for training and skills building. Difficulties encountered along the way were taken seriously, and good and secure solutions were found through joint efforts. Support from the leadership was experienced as thoroughgoing, there was a clear sense that the work was both important and meaningful.

Systems interventions were carried out to secure resources and support, as well as making the MFT work itself visible, both internally in RESSP and externally to the clinic leadership with thought to the professional and economic support needed. More widely to NKNS, for example, in order to spread knowledge of and experience of the MFT work.

Decision support and data systems were put in place. Regular meetings were held with the therapists who gave verbal feedback about their experience with the work. With the final form of the model in mind, an evaluation was made of each group gathering, as well as after the end of the whole MFT group programme. The evaluation was valuable in respect of the therapists’ commitment to, and willingness to stay with, the MFT work.

The quality of implementation was also assessed. When looking at what has been done, there is no apparent discrepancy between what was originally planned and what has, in fact, been achieved. The work has been carried out and an MFT model developed and integrated as a permanent service within RESSP. There has been a thoroughgoing support system. Factors which have supported the enterprise have been training and skills building, supervision and ongoing evaluation, together with that space has been made among the everyday clinical work for the work to be carried out.

### How many have taken part?

The first MFT group was held in 2007. Between 2007 and 2018 there have been 11 groups. In each group there have been between 5 and 8 patients, together with their family members. On average, each family has comprised 4 members. In total, 68 patients have entered the MFT process. The number of participating family members is 198, of which 65 mothers, 56 fathers, 59 siblings (35 sisters and 24 brothers), 12 partners/boy−/girlfriends and 6 other close people – parent’s partner/spouse, other relatives (grandparents) and friends. (Siblings do not usually come to all of the meetings, but 50 have been more than once outside of the sibling gathering.)

All the service users have been women. The average age at group start was 21.3 ± 3.5 (mean ± SD) (median 20.1; range 17.1–30.1) (*N* = 68). The largest number of the participants, 52 (76.5%) had a diagnosis of anorexia (AN) while 16 (23.5%) had a diagnosis of bulimia (BN). Age at start for the anorexia patients was 20.7 ± 3.1 (median 20.0; range 17.1–30.1) (*n* = 52), and for those with bulimia 23.3 ± 3.9 (median 23.1; range 18.1–30.1) (*n* = 16). Of these participants, 42 (61.8%) belonged to Nordland county, 22 (32.4%) to Troms and 4 (5.9%) Finnmark.

Of the 68 who started, 63 completed the course of treatment, giving a dropout rate of 7.4%. Of the 5 who dropped out, 3 had a diagnosis of anorexia and 2 bulimia. Of these, 3 were from Nordland and 1 each from Troms and Finnmark.

BMI at start was 17.8 ± 2.1 (mean ± SD) (median 18.2; range 13.5–21.9) (*N* = 66; missing = 2). BMI for those with AN (*n* = 52) was, at start, 17.2 ± 1.9 (median 17.5; range 13.5–21.2), and for those with BN (*n* = 14; missing = 2) was, at start*,* 20.2 ± 1.1 (mean ± SD) (median 20.5; range 17.9–21.9).

Those who completed the programme had, at start, a BMI of 17.8 ± 2.1 (mean ± SD) (median 18.1; range 13.5–21.9) (*N* = 62; missing = 1). And, by the end, a BMI of 18.0 ± 2.0 (mean ± SD) (median 18.0; range 13.7–22.2) (*N* = 55; missing = 8). There was not a significant weight change at the end of treatment (BMI difference of − 0.38 ± 1.9 kg/m^2^, range − 0.89 – 0.13 kg/m^2^, *p* = 0.141). The effect size for this change was negligible (Cohen’s d = 0.1).

Regarding patients with a diagnosis of anorexia, at start they had a BMI of 17.2 ± 1.9 (mean ± SD) (median 17.4; range 13.5–21.2 (*n* = 49)*.* By the end this group had a BMI of 17.7 ± 2.0 (mean ± SD) (median 17.5; range 13.7–21.5) (*n* = 46; missing = 3). There was not a significant weight change at the end of treatment (BMI difference of − 0.54 ± 1.9 kg/m^2^, range − 1.1-0.03 kg/m^2^, *p* = 0.062). The effect size for this change was small (Cohen’s d = 0.26).

Looking specifically at those with an underweight at start, defined as a BMI below 18.5, 42 (63.6%) (*N* = 66; missing = 2) had a BMI below 18.5. Of these, 40 (95.2%) had a diagnosis of anorexia and 2 (4.8%) bulimia. Their average age at start was 20.7 ± 3.2 (median 20.0; range 17.1–30.1) (*n* = 42). They had a BMI at start of 16.6 ± 1.5 (mean ± SD) (median 17.0; range 13.5–18.4) (*N* = 42). Those who completed the programme had, at the start, a BMI of 16.6 ± 1.5 (mean ± SD) (median 17.0; range 13.5–18.4) (*N* = 40). Of theses, 38 (95%) with AN and 2 (5%) BN. By the end the BMI was 17.4 ± 1.9 (mean ± SD) (median 17.5; range 13.7–21.5) (*N* = 37; missing = 3). There was a significant weight change at the end of treatment (BMI difference of − 0.88 ± 1.9 kg/m^2^, range − 1.50 - -0.25 kg/m^2^, *p* = 0.007). The effect size for this was medium (Cohen’s d = 0.48).

Of those completing the programme, 56 (88.9%) were evaluated as being in need of further treatment, whilst 2 did not (don’t know = 5). Of these 56, 54 have continued in treatment – that is, the great majority (96.4%) of the patients completing the programme.

### Dissemination, and training of other professionals

A training module was developed in order to disseminate the experience and expertise gathered by means of the MFT practice at RESSP. This was targeted at professionals working at other Norwegian treatment centres for eating disorders. The user-organisations ROS and SPISFO were also invited. This service received financial support from the Norwegian National Clinical Network for Eating Disorders (NKNS).

### Objectives and framework

Beyond introducing others to the MFT work, it was of high value to us that this way of working be seen as useful, something that could be taken into the therapeutic work of these participants.

The course began in autumn 2015 and ran over eighteen months. It comprised 6 meetings, one of 3 days, the others each 2 – the same as with the clinical MFT work. There were 32 participants from other specialist regional eating disorder centres, local eating disorder treatment centres, child and adolescent and district psychiatric centres, together with ROS, a user organisation. (RESSP: 7, BUPA, Nordland Hospital: 4, DPS Nordland Hospital: 2, the regional centre in Levanger: 7, ‘Eating-team’, Vestfold, Tønsberg: 4, Modum Bad: 1, Vestre Viken, Drammen: 5, ROS: 2). The group comprised the following: 16 nurses, 8 psychologists, 2 psychiatrists, 1 social worker, 1 clinical educator, 2 art therapists and 2 others. Those responsible for the training were 3 highly experienced therapists who had been involved in the MFT work from the first.

### Structure and content of the gatherings – themes

The structure of the gatherings followed the group-based form of the MFT service, and a practical approach to sharing knowledge and experience was chosen. To give the professionals the best possible experience of the method, they were divided into 6 ‘families’ throughout the training. As well as being ‘family members’, they would perform the role of therapist. As in the MFT groups, the gatherings moved between different group settings with large and small groups and space for group discussion and supervision. Of the 6 gatherings, 3 addressed communication, sibling-gathering and ‘mind the gaps’. Other essential themes taken up were: role of the therapist, how to ‘read a group’, how the therapist is affected, challenging situations and the use of different interventions. These were introduced first by means of verbal presentations, as in the clinical programme. Group members had responsibility for presenting some of the issues from out of pre-available information.

### Therapeutic methods and group processes

Different interventions are used to make contact with, engage and mobilise each group member, and the group as a whole. There is also the question of eliciting the resources of both individuals and group. Different kind of interventions are used to regulate the emotional climate in the group and to manage the different topics. It was crucial to teach how these interventions could be used in group therapeutic work. In order to teach and to train in the use of these, practical exercises were included in the groups. The group members were invited to lead some of these exercises. These methods were comparable to those used in the MFT model.

It was fundamental to show how to work with the processes arising in the group and how to follow these processes and respond to them. In this, it was vital that the participants were able to develop an awareness of positive and negative emotionality in the group, to register changes in it, and to move between following the laid-down programme and responding to those changes actively and in real time.

### Feedback and evaluation

An evaluation was carried out after each gathering, as well as at the end of the training. Feedback on the following points was given on a 5-point scale: organisation and leadership, aims and content (with respect to usefulness) as well as presentation, delivery and an overall score. Additionally, there was an evaluation of the whole of each gathering. (During the final gathering there was also a creative evaluation of the training in which each ‘family’ made a picture that summarised their experience of it.) The feedback received was that the processes that had been gone through had been inspirational and useful. The final evaluation gave the following results: organisation and leadership 4.68 ± 0.6 (mean ± SD) (median 5.00; range 3–5), objectives and content 4.76 ± 0.5 (median 5.00; range 3–5), presentation and approach 4.8 ± 0.4 (median 5.00; range 4–5), and as a whole 4.8 ± 0.4 (median 5.00; range 4–5).

### Outcome of the training

By the end of the training, 3 of the participating units - Levanger (where their second group was underway), Tønsberg and Drammen - had established their own MFT groups.

## Discussion

The primary aim of the project was to establish an MFT service for young adults with a severe eating disorder at RESSP.

### Implementation of the MFT service – design of an MFT model for young adults and experience of its implementation

The work to develop an MFT service for young adults and their family members took place over time, and after a 3-year trial period a model was arrived at that has essentially been retained since. This model comprises 6 group-based gatherings over a 12-month period, totalling 13 days. The model is fully described in its handbook [55].

The model for young adults has a different aim to that for children and young people; involving that the young adults take responsibility for their own lives and health, a natural process toward independence. Accordingly, the structure of the gatherings, content, themes and interventions were adjusted to the age of young adults.

Compared to the models from London and Toronto, this model is similar to the one for children and adolescents in that it is comprehensive, gathering-based and runs over a longer period. This service can be said to represent a new way of organising the treatment of young adults with severe eating disorders. What is new is that the patient’s family is actively involved in a treatment programme.

Including family members and others close to the patient in the treatment, together with other families creates an arena for work with essential and relevant topics by means of psychoeducation and in various group settings. In an MFT group, the therapist has more to work with, and the dynamic group processes involved lead to new openings for contribution, challenge and interplay. The objective is to provide the best possibility for recovery in which ‘playing as a team’ is a vital feature, and where unhelpful patterns around communication, interaction and relating in a constructive way are made apparent [27, 44–46].

It is crucial to understand how the eating disorder plays itself out and how it can overwhelm interactions within the family. The family members need to be aware of how they can make a positive contribution to the treatment, and that the patient be aware of their own responsibilities.

By involving the families, a dynamic is created where significant issues can actively and creatively be explored and the space found for new understanding and change – something which gives a new starting point for handling difficulties, stresses and emotional responses.

The model creates an arena where it is possible to meet with and talk to other families in a similar situation, and thereby experience being understood and recognised. Many have experience of isolation and stigmatisation, things that can be spoken about with the other participants. The group is a place in which many of the patients function quite well and show other sides of themselves than is usual in a traditional treatment setting. It can be the case that MFT addresses the healthy part of the person, giving it room to emerge.

MFT is a form which fosters change and growth. It is sufficiently engaging and involving that the participants take an active part in it. It might be reasonable to suggest the applicability of the MFT model for other clinical groups.

### Implementation of the change needed to establish an MFT service for young adults at RESSP

The process of change comprises several phases [47–49]. What marks out this process at RESSP is that from the first the staff were actively involved and had a crucial role in the development work. They took ownership of ‘the project’, with a lasting sustained loyalty, despite the difficulties involved, this is supported by other studies [52]. The developments and changes were based on a shared understanding and aspiration for their achievement, together with clear prioritisation from the leadership. Studies in the field of implementation research show that the best starting point is where the initiative for change comes from both the staff (‘bottom up’) and the leadership (‘top down’) [49, 56]. Change is demanding and complex and success depends on both a good foundation from the staff and support from the management. Releasing sufficient resources, amidst a busy clinic environment, for training, supervision and to stand by the developmental work was vital in enabling the project of development and change. The MFT service was found to be a significant addition to the treatment offered by RESSP, accommodating the desire to include family members and others close to the patients in the treatment. The MFT model is experienced as an improvement on what had been offered previously. The model has been followed throughout subsequent years and experienced as sustainable, now in place as a permanent service within the unit.

### Extent of the innovation – key components of the completed development

The practicalities of implementation are described in terms of the core components sketched out by Fixsen et al. [50]. In summary, this is characterised by the way in which the therapists at RESSP were invited to share in the development work and change-making by the leadership who had a clear wish that the changes be effected. A multidisciplinary team was formed and time set aside for training, skills acquisition, and supervision from experienced MFT therapists. Provision was made for ongoing feedback and evaluation from the therapists which was crucial with respect to the evolution of the therapist role, and the interplay between the different therapists. Ongoing evaluation was also applied to the different components of the innovation in order to be sure that all of the different elements in the process were attended to. The leadership supported and secured the process, and the work was done in an open and visible way in order to obtain both professional and economic support. Both, group members and therapists contributed ongoing evaluation of the decisions made in connection with the design of and the conduct of the model.

Introducing the new service was a significant decision for the regional centre. A decisive feature was the engagement of the staff and leadership. The leadership was willing to give both priority and resources to the implementation work [47, 53]. Another feature was that time was set aside sufficient to allow for follow up and evaluation of both the therapists’ experience of, and the implementation of, the process itself - something that gave a necessary underpinning to the work involved. Support from elsewhere within the hospital was a requisite, providing economic security. Continuous feedback from therapists and participants was a valuable and necessary element in the shaping of the model.

The quality of the implementation can be considered as good, in that there was a good match between what was originally planned and what was actually done. The process was well supported so that the MFT model was established as a permanent service at the unit.

### How many have been involved?

The first MFT group began in 2007, and up to now there have been 11 groups with 68 young, adult female patients and 198 family members, including mothers, fathers, siblings and others close to the patients.

The average age of the patients at start was 21.3 years and the majority (76.5%) had a diagnosis of anorexia, the others (23.5%) bulimia. Most of the patients (61.8%) came from Nordland county, and fewest from Finnmark (5.9%). Of 68 patients, 63 completed the MFT programme (dropout 7.4%). Average BMI for the whole group at start was 17.8, 17.2 for those with anorexia and 20.2 for bulimia. At start 42 (63.6%) of the patients were underweight. Change in BMI between group start and end was significant for those being underweight at start. Of the 63 completing the programme, the majority (*n* = 56) was seen as needing further follow up and treatment: 54 of these 56 have continued in treatment.

Both patients and their families were interested in participating in the MFT service and readily accepted the offer to join. The majority of the patients are young women in their early twenties with a diagnosis of anorexia. Anorexia is a condition requiring treatment over many years and which powerfully affects others in the family [11–16]. MFT is therefore of high value to this group because of the opportunity to look closely at interplay and relationships within the family and how best to support the process of recovery.

A significant outcome is that 63 patients out of 68, and their families, completed the MFT process. In the context of eating disorders this is a strikingly low dropout rate. Studies show a consistently high proportion of patients leaving their treatment programme [18, 21, 23]. One of the main challenges in the treatment of eating disorders is that of retaining the patient in the treatment. The new service has shown itself to be sufficiently interesting that the patients stay with it. The patients have, in addition to the MFT, follow up from their own local therapist, which also helps in working one’s way out of the eating disorder.

This MFT programme doesn’t work specifically with weight gain as an objective, but measurements of BMI show that weight goes significantly in the right direction for those with an underweight at start.

Having gained sufficient self understanding to acknowledge their need for further treatment, the majority of those requiring further follow up and treatment, continue to stay in it. Remaining in treatment for an extended period is called for and increases the possibility that the patient can later take responsibility for their own recovery. It therefore appears that including family members and others close to the patient in the treatment can help young adult patients to engage positively in, and stay in, a necessary treatment process.

To document the usefulness of the MFT treatment, a research project was initiated in 2015 measuring outcomes using values recorded at start and end of the process. In addition to BMI values, among others socio-demographic data are collected, changes in eating disorder symptom load and functionality, quality of life, as well as changes in experienced unity and communication within the family.

### Dissemination, and training of other professionals in Norway

A training programme for 32 diverse professionals from other treatment centres in Norway, together with representatives of the user organisation ROS was planned, developed and delivered. The training was arranged also out of a wish among others to learn about the MFT treatment model. The training ran over the course of eighteen months and was structured in much the same way as the clinical MFT programme.

It was an objective that the training be useful and something that could be used in other centres. The training programme received good feedback. By the end of the training, 3 of the centres had begun their own MFT groups for young adult patients.

The interest taken in the training, and the level of participation in it, evidenced that there was a need for, and a wish to involve families in the treatment of young adults. There was considerable interest in treatment lending itself to the inclusion of family members and others close to the patients.

The positive feedback and the initiation of MFT groups elsewhere showed the benefit of the new model. Its practical approach, leaving space for demonstration and exercises, together with supervision made it reasonable to start similar services at the different treatment centres.

## Conclusion

The main aim of the work was to establish at RESSP an MFT service for young adults with a severe eating disorder. A process of development and of change was set in motion. This work was carried out according to implementation theory and was achieved by means of a long process of systematic, planned innovatory work. This had active, participatory support from the unit leadership, and was founded on the engagement and loyalty of the staff. As a result, a model was developed and a service established, meeting the original wish to include families in the treatment, and this was gradually integrated as a permanent service.

The service was of interest to patients and families. It has had a strikingly low dropout rate, and most patients have continued their treatment, in itself an important objective. Measuring BMI showed a significant weight-gain for those with an underweight at start. It therefore appears that including family members and others close to the patient in the treatment can help young adult patients to engage positively in and stay in a necessary treatment process.

The MFT model can be applicable to work with other mental disorders where the participation of family members is desirable.

Other professionals from different professional settings throughout the country were interested in the training programme in MFT. The model was introduced in other treatment centres.

A research project was initiated to document and elaborate on the usefulness of the MFT treatment. This project began in 2015 and comprises both qualitative and quantitative measures of outcomes using values recorded at start and conclusion of the process. It is intended that further papers will come from this.

## Data Availability

Please contact author for data requests.

## Abbreviations

AN: Anorexia nervosa
BMI: Body mass index
BN: Bulimia nervosa
BUPA: Department of Child and Adolescent Psychiatry
DPS: Local Psychiatric Centre
FDS: Family Dialogue Set
MFT: Multifamily therapy
NKNS: National Clinical Network for Eating Disorders
NLSH: Nordland Hospital
RESSP: Regional Centre for Eating Disorders
ROS: Eating Disorder Counselling Service
RSS: Regional Centre for Eating Disorders
SD: Standard deviation
SPISFO: The Eating Disorder Association
